# Local self-uniformity in photonic networks

**DOI:** 10.1038/ncomms14439

**Published:** 2017-02-17

**Authors:** Steven R. Sellers, Weining Man, Shervin Sahba, Marian Florescu

**Affiliations:** 1 Advanced Technology Institute and Department of Physics, University of Surrey, Guildford GU2 7XH, UK; 2 Department of Physics and Astronomy, San Francisco State University, 1600 Holloway Avenue, San Francisco, California 94132, USA

## Abstract

The interaction of a material with light is intimately related to its wavelength-scale structure. Simple connections between structure and optical response empower us with essential intuition to engineer complex optical functionalities. Here we develop local self-uniformity (LSU) as a measure of a random network's internal structural similarity, ranking networks on a continuous scale from crystalline, through glassy intermediate states, to chaotic configurations. We demonstrate that complete photonic bandgap structures possess substantial LSU and validate LSU's importance in gap formation through design of amorphous gyroid structures. Amorphous gyroid samples are fabricated via three-dimensional ceramic printing and the bandgaps experimentally verified. We explore also the wing-scale structuring in the butterfly *Pseudolycaena marsyas* and show that it possesses substantial amorphous gyroid character, demonstrating the subtle order achieved by evolutionary optimization and the possibility of an amorphous gyroid's self-assembly.

## Abstract (web summary)

The interaction between photonic bandgap materials and light is largely determined by the wavelength-scale material structure. Here, Sellers *et al*. develop a new metric of network structural order and demonstrate its connection to the photonic bandgap of an amorphous gyroid network.

A complete photonic bandgap (PBG) is frequency window within which a material, by virtue of its structure, supports no propagating electromagnetic modes. Typically, structures which possess complete PBGs are periodic arrays of dielectric material; such arrays are called photonic crystals (PhCs). PhCs have the potential to play a key role in the development of next-generation photonic integrated circuits^1^,^2^,^3^. However, although the complexity of PhC-based technologies continues to grow, questions regarding the fundamental mechanisms of PBG formation remain unresolved^4^,^5^,^6^.

The formation of PBGs is conventionally interpreted as a result of coherent scattering by a PhC's lattice planes^7^,^8^. In this picture, a plane wave may be scattered onto its counter-propagating equivalent when the wavevector of the initial state lies on the edge of the PhC's Brillouin zone (BZ). When this condition is met, a pair of orthogonal standing wave modes, each possessing a distinct electromagnetic field profile, is formed^5^,^9^,^10^. Energetic interaction between the electric field and the underlying dielectric distribution then breaks the degeneracy of the standing wave states. For the specific propagation direction under consideration, the resulting forbidden spectral range defines a photonic stop gap.

To engineer a complete PBG, photonic stop gaps must open along all propagation directions. Further, these stops gaps must be spectrally aligned. Both these considerations can be addressed by designing PhCs to possess maximally spherical BZs^11^. The search for the first complete PBG thus focussed on face-centred cubic crystals—the most isotropic of the three-dimensional (3D) Bravais lattices—and discovered a large complete gap in a diamond-like network of dielectric material^12^. In spite of the many PhC designs that have since been discovered, those based on the diamond network remain the champion, possessing the largest complete PBGs^13^,^14^.

There is, however, much evidence to suggest that PBG formation is governed by more than just coherent scattering processes. PhCs derived from the body-centred cubic single-network gyroid (SNG) structure (triamond) and low-symmetry diamond embeddings all possess near-champion PBGs in spite of their less spherical BZs^13^. Although based on a face-centred cubic lattice, the inverse opal network exhibits a complete PBG only one quarter the size of the champion diamond gap^1^. Most tellingly, a glassy 3D network—dubbed photonic amorphous diamond (PAD)—exhibits a sizeable complete PBG^4^. This gap exists despite PAD's diffuse primary diffraction maximum which spreads the structure's coherent scattering power of a range of wavevectors^5^.

Similar evidence is found in two-dimensional structures that possess PBGs for light with a transverse electric (TE) polarization. Amongst these structures, the champion PhC design is a honeycomb network of dielectric material^15^. Many glassy networks that can be broadly styled as ‘hyperuniform disordered honeycombs' have been found to possess sizeable TE PBGs^6^,^16^,^17^,^18^,^19^,^20^. As with PAD, these gaps exist despite the diminished coherent scattering power of each structure.

Here, we address the mechanisms governing PBG formation by re-formulating the ideal structural properties of a PBG-forming network. To achieve this, we introduce the concept of local self-uniformity (LSU). LSU measures the geometrical and topological similarities of the local environments in a connected network of uniform valency. We note that existing sizeable PBG networks possess significant LSU. We demonstrate the connection between LSU and PBG forming ability by designing novel amorphous gyroid (amorphous triamond) connected networks. Specifically, amorphous gyroids can possess sizeable PBGs and an amorphous gyroid's LSU is strongly correlated with its PBG width. This correlation is explained by recognizing the contribution of spatially localized resonant scattering processes to PBG formation in connected networks. Locally self-uniform ceramic 3D-printed amorphous gyroids are characterized through microwave transmission experiments. We explore also the possibility that amorphous gyroid exists within the wing scales of butterflies. In particular, we reveal that the microstructure in the scales of *Pseudolycaena marsyas* possesses substantial amorphous gyroid character and demonstrate that the butterfly's reflectance spectrum can be effectively reproduced by amorphous gyroid microstructures.

## Results

### Local self-uniformity

The exact structure of glasses has long been debated^21^. Recent research has demonstrated the complex interplay of ordered and disordered phases in the vitreous state^22^,^23^,^24^. The disparate variety of bulk metallic glasses in particular has challenged researchers to develop predictive theories of an alloy's glass-forming ability^25^. The existence of sizeable PBGs in glassy networks exposes an analogous deficiency in current understanding of a structure's PBG forming ability. Unlike silicate and metallic glasses, the structures of glassy PBG materials are designed. Nevertheless, the structural characteristics that render these glasses amenable to PBG formation remain mysterious. With the aim of clarifying these PBG forming characteristics, here we develop LSU a general measure of structural order in connected networks.

**Figure 1:**
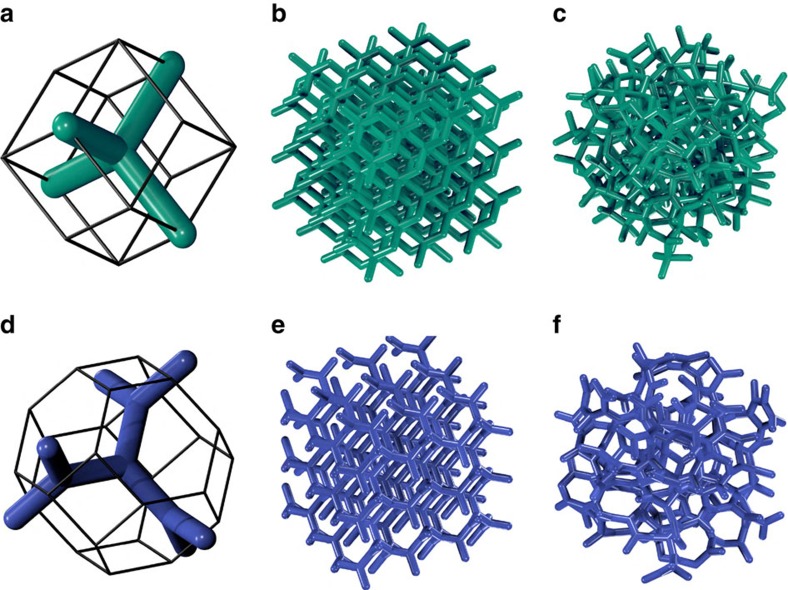
Strongly isotropic networks and their amorphous derivatives in three dimensions. The WS cell of a diamond PhC (**a**) contains a 1-tree of dielectric material (green); the edges of the tree define a tetrahedral unit. Stacking of the WS cells generates the champion diamond PhC (**b**). Amorphous diamond (**c**) consists of deformed tetrahedral units connected in a CRN. Similarly, the WS cell of a single-network gyroid PhC contains a 2-tree (**d**), comprising dielectric material (blue), in which each vertex is a trihedron. When stacked, the WS cells generate a single-network gyroid PhC with a near-champion PBG (**e**). Amorphous gyroid (**f**) comprises deformed trihedral units connected in a CRN.

A typical PhC consists of a connected distribution of dielectric material surrounded by air. As an example, Fig. 1a,b show the champion PhC diamond; it is a connected network of dielectric cylinders (green) arranged as in a diamond crystal. A PBG's size is usually measured as a dimensionless width given by Δ*ω*/*ω*_0_—the frequency width divided by the central frequency. Figure 1c displays an amorphous version of the diamond network. The complete PBG in diamond has been shown to have a width of 30% for dielectric material of refractive index 3.6 (ref. 13). We focus our discussion of PBG properties hereafter in this high-refractive index regime.

To describe a general connected network, we decompose it into a set of vertices and edges. A vertex is a point at which two or more distinct lobes of material intersect. An edge is a vector between two vertices that specifies the central axis of a lobe of material. As an illustration, Fig. 1a shows the fundamental unit of a diamond network in its Wigner–Seitz (WS) cell. A vertex, which sits at the centre of the cell, has four edges connecting it to its nearest neighbour vertices. The four nearest neighbours sit at the corners of a tetrahedron and the four edges define a tetrahedral unit. Similarly, Fig. 1d shows the fundamental unit of a SNG structure in its WS cell. The central vertex has three nearest neighbour vertices to which it is connected by three edges. These edges are of equal length and are separated by inter-edge angles of 120°; we call this configuration a trihedral unit.

The WS cell is the basic building block from which an extended periodic network can be assembled. Stacking the WS cells of diamond and SNG produces extended diamond (Fig. 1b) and gyroid (Fig. 1e) networks, respectively. An extended periodic network of this type is a highly ordered case of a continuous random network (CRN). A general CRN is a collection of vertices, connected by edges, which are arbitrarily positioned in space^26^. CRNs do not typically possess translational periodicities as the diamond and gyroid crystals do. Instead, Fig. 1c illustrates a classic CRN—an amorphous diamond network, studied extensively as a model of amorphous silicon^27^,^28^. Every vertex in amorphous diamond is tetravalent and has four nearest neighbours that describe the points of a deformed tetrahedron. The resulting network is a connected assembly of deformed tetrahedral units. Similarly, an amorphous gyroid is a CRN comprising deformed trihedral units as depicted in Fig. 1f.

We now introduce the concept of a tree within a CRN. An *n*-tree on a vertex *a* of a CRN is the set of edges and vertices that can be reached from *a* by traversing no more than *n* edges. The tree's ‘root edges' are understood to mean the edges belonging to vertex *a*. To illustrate this, the diamond WS cell (Fig. 1a) contains a 1-tree. The SNG WS cell (Fig. 1d) contains a single 2-tree. A tree thus describes a small network unit within a CRN.

We define LSU as a property of a CRN that describes the extent to which its component *n*-trees have shapes similar to that of nearby *n*-trees within the network. For the moment, we consider comparing some example 1-trees. Such trees consist of a central vertex with a number of edges *γ*. At first, we consider two trihedral 1-trees which we label 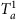 and 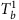.

**Figure 2:**
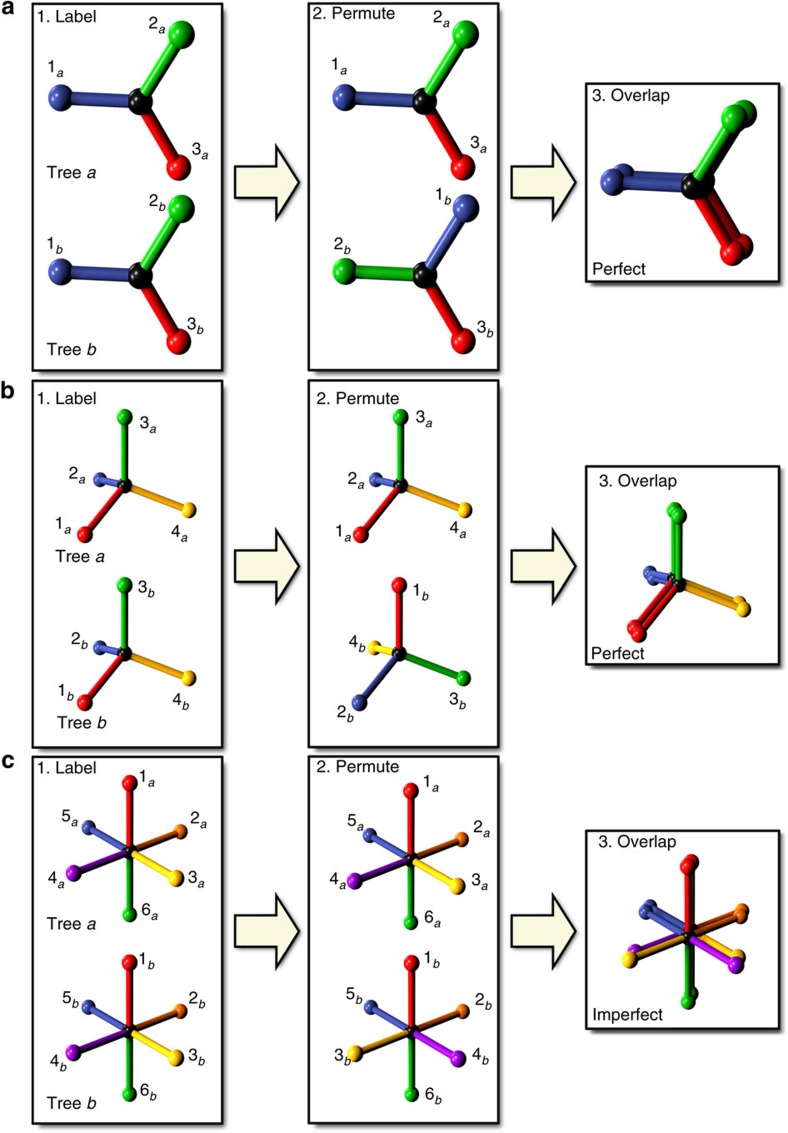
Comparison of 1-trees illustrated with trihedra, tetrahedra and octahedra. Two identical trihedral trees (**a**) are labelled by their edges. The edges of tree *b* are then permuted. Tree *b* can then be rotated around the edge 3_*b*_ axis and made to perfectly overlap tree *a*. Similarly, two identical tetrahedral trees are labelled (**b**), and then the edges of tree *b* permuted. Reflection of tree B in the plane of edges 2_*b*_ and 4_*b*_, followed by rotation around the new edge 3_*b*_ axis, brings the two trees into alignment. Two identical octahedral trees (**c**) can also be compared. We apply a permutation to tree B's edges. When overlapped, the two trees are now mismatched; their yellow and purple edges are not aligned and cannot be made so by any congruent transformation without creating a new mismatch.

To measure the extent to which these two trees have similar shape we follow the process shown in Fig. 2a. First, we label the root edges of both trees as (1_*a*_, 2_*a*_, 3_*a*_) and (1_*b*_, 2_*b*_, 3_*b*_). Second, we specify a permutation of the labels of tree *b*, say [213]. Third, we attempt to align the two trees such that the root edges overlap in the prescribed permutation. This alignment is achieved by performing a congruent transformation on tree 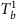. That is, we physically pick up the tree and perform any combination of a translation, a rotation and a reflection until the edges of both trees overlap as desired^29^. Fourth, we score the extent to which 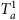 and 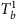 now overlap by taking the scalar product of the edge vectors in pairs as determined by the permutation. The result of this comparison is normalized such that perfectly overlapping trees receive a maximum score of 1.

Finally we repeat steps 1 through 4 and score the overlap of 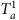 and 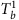 for all *γ*! permutations of their root-edge alignment. We define the spatial similarity statistic *φ*_*ab*_ of these two trees as the average result of the *γ*! comparisons (see the Methods section).

This process can be applied without modification to determine the spatial similarity statistic of two identical tetrahedral 1-trees, as shown in Fig. 2b. As before, we label the root edges of the two trees and specify some arbitrary permutation of the root edges of tree *b*; Fig. 2b shows the permutation [2413]. Once permuted, tree *b* is congruently transformed by a translation, a reflection and a rotation to maximally overlap with tree *a*. The quality of the overlap is then measured. The average overlap quality for all 4! root-edge alignment permutations defines the spatial similarity statistic *φ*_*ab*_.

In Fig. 2a,b, we note that that the two trihedra and tetrahedra could be made to overlap exactly for their respective root edge permutations. In fact, pairs of either trihedra or tetrahedra will overlap exactly for all root edge permutations and have a spatial similarity statistic of unity. This property of trihedral and tetrahedral units is a corollary of work on strong isotropy^29^. There exist only three strongly isotropic networks in three dimensions or less. In three dimensions, these networks are diamond (Fig. 1b) and single gyroid (Fig. 1e). In two dimensions, the honeycomb network is the only strongly isotropic network.

We consider now a third set of example 1-trees—two identical simple cubic network units (Fig. 2c), each with six edges about a central vertex—and calculate their spatial similarity statistic. We find that perfect overlap can be achieved for specific root edge permutations, but in most cases there is no congruent transformation to align the two trees correctly; we call such a permutation inaccessible (this is depicted in Fig. 2c). As a result, the simple cubic crystal does not possess a strong isotropy property. In the remainder of this paper, we focus on trees comprising trivalent and tetravalent vertices only.

To build a robust measure of network structural order, we require a formalism that can compare the trees of arbitrarily disordered networks. We thus build upon the measurement of tree spatial similarity statistics in the following ways. We stipulate that the CRN from which the trees are drawn comprises only vertices with a fixed number of edges. We then generalize the measurement of tree spatial similarities to *n*-trees of any size. In this case, the correct edge overlap pairings are not known beyond the root edges. To solve this, we have developed an algorithm which determines the natural edge overlaps; this is detailed in the Methods section. We also allow the similarities of arbitrarily disordered trees to be measured. Here we acknowledge that the root edges of two trees will not necessarily overlap perfectly. Instead, for each permutation we find a transformation that yields an approximate overlap. We then quantify the quality of this overlap according to a metric of spatial similarity (see Methods section).

We now define a CRN's LSU distribution Φ_*nl*_ to depth *n* and locality *l* as the set of *n*-tree spatial similarities *φ*_*ab*_ for all pairs of trees whose root vertices are within *l* edges of one another. The LSU distribution is a set of spatial similarities that describes the extent to which the CRN's geometry is uniform on the length-scale *l*. For example, Φ_22_ describes the spatial similarities for all pairs of trees of depth 2 separated by two edges or less.

**Figure 3:**
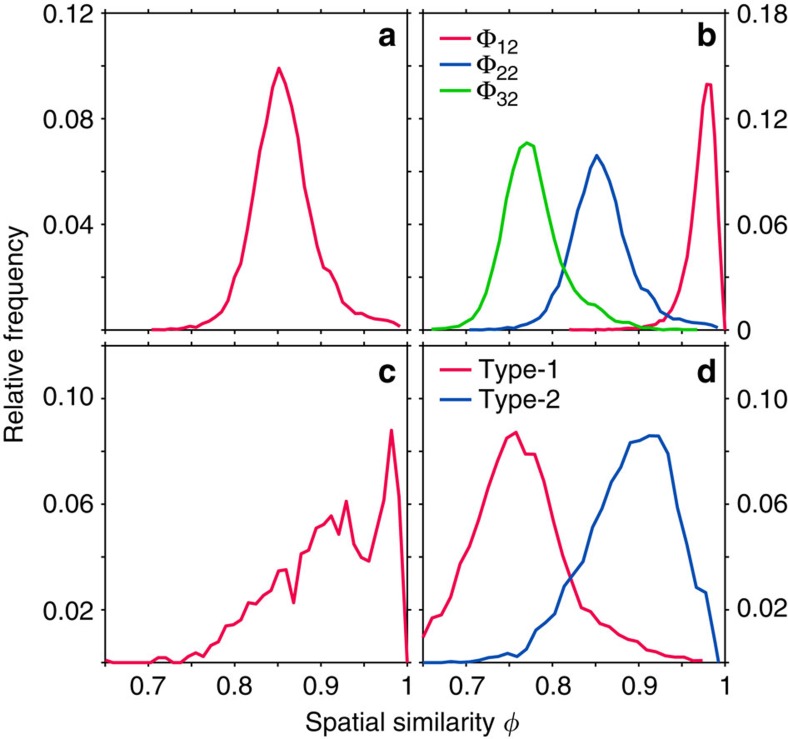
Example LSU distributions. (**a**) The distribution of spatial similarity statistics for tree comparisons of depth 2 and locality 2 (Φ_22_) for an amorphous diamond network. (**b**) LSU distributions Φ_*n*2_ of amorphous diamond for different tree depths *n*. (**c**) Φ_22_ for a 216-vertex sample comprising a single gyroid crystallite suspended in amorphous gyroid, demonstrating LSU's ability to differentiate between phases. (**d**) Φ_22_ distributions for two characteristic types of amorphous gyroid network—type-1 (1-tree local order) and type-2 (2-tree local order).

We present a set of example LSU distributions in Fig. 3. Figure 3a shows Φ_22_ for an amorphous diamond network. The trees in amorphous diamond are non-identical and present a broad distribution of spatial similarity statistics. However, the spatial similarities remain relatively large and show that the local geometries in the CRN are similar. Fig. 3b compares three LSU distributions, Φ_12_, Φ_22_ and Φ_32_, for amorphous diamond. Amorphous networks have strong positional correlations at local length-scales that fade with distance; as a result, trees become more spatially dissimilar as their depths increase. We note that strongly isotropic networks are the only CRNs whose Φ_*nl*_ comprise a single peak at unity for all tree depths and localities; they are uniquely identified by their LSU distributions. This property is a consequence of the definition of strong isotropy^29^. The LSU distributions are thus naturally interpreted as a continuous measure of the extent to which a network is strongly isotropic.

LSU distributions can also be used as a diagnostic of the phases present in a CRN. Figure 3c presents Φ_22_ for an amorphous gyroid sample containing a gyroid crystallite surrounded by an amorphous network. The distribution comprises a background spectrum from which a clear peak emerges ∼*φ*=0.98. The peak signifies the existence of network regions comprising a strained crystalline gyroid phase. The LSU distribution is thus diagnostic of separate phases within a system and is useful for probing the local order of complex systems undergoing phase transitions^22^.

### Champion photonic bandgap architectures

3D architectures which exhibit large complete PBGs, together with two-dimensional architectures with large TE PBGs, possess a number of shared structural characteristics. First, successful architectures are connected networks of dielectric material^5^,^18^,^30^. Second, the champion structures—the honeycomb and diamond networks in two and three dimensions, respectively—are both strongly isotropic. SNG, which is the only other strongly isotropic network, exhibits a near-champion PBG of width 28% for a refractive index contrast of 3.6:1 (see Supplementary Fig. 1). Third, amorphous derivatives of both honeycomb and diamond networks exhibit PBGs which are sizeable but smaller than the gaps of their parent crystalline networks. Crucially, both networks possess significant but imperfect LSU (see Fig. 3a).

Overall, evidence suggests that the extent of a network's LSU influences its PBG forming ability. Hence, we expect that a hypothetical amorphous SNG, analogous to PAD^4^ and disordered honeycomb^6^, should possess both a high degree of LSU and a sizeable complete PBG. To the best of our knowledge, amorphous gyroids have not been observed in any context. We thus undertake the design of amorphous gyroids as a means of testing the relationship between LSU and PBG forming ability. To achieve this, we apply the Wooten-Winer-Weaire (WWW) algorithm to anneal amorphous gyroids from random seed networks^27^,^28^. In order to yield faithfully gyroidal local geometries, networks are annealed using a modified potential energy function that is distinct to the regular Keating energy^27^ (see also Supplementary Methods).

We generated a set of amorphous gyroid models of varying total size as measured by their number of component vertices *N*. In particular, we generated an ensemble of 57, 216-vertex models across a spectrum of disorder. PBGs were probed by numerical solution of the Maxwell equations via a plane wave expansion method^31^. A refractive index contrast of 3.6:1 was used for all calculations. Bandgap widths were measured as a percentage of their central frequency.

We demonstrate that amorphous gyroid networks can possess sizeable PBGs. The average PBG measured for a set of high-quality 1,000-vertex networks was 16%. The largest single gap observed was for a well-annealed 216-vertex network and had a size of 21%. These gap widths compare favourably with the 18% gap in PAD at the same refractive index contrast^4^.

**Figure 4:**
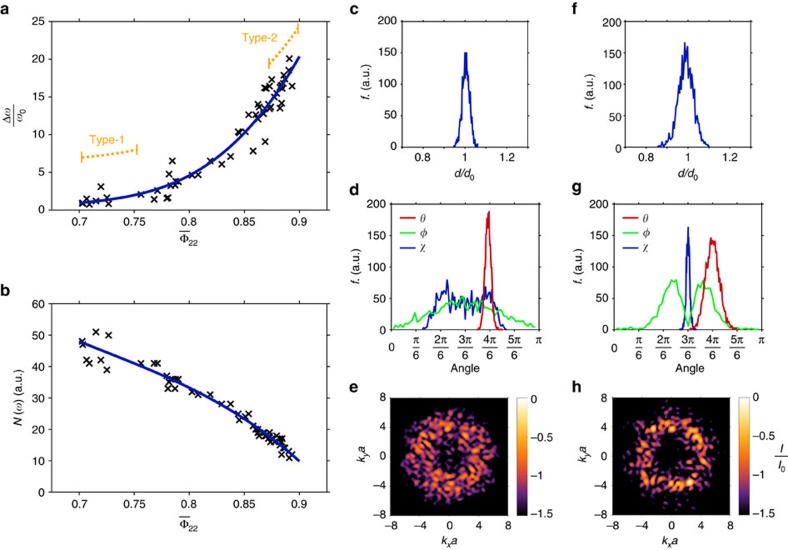
LSU and PBG forming ability. The LSU of amorphous gyroid networks, as measured by the mean of their Φ_22_ distributions, is strongly correlated with PBG width (**a**). Similarly, the integrated DOS *N*(*ω*) decreases smoothly with increasing Φ_22_ (**b**). Fit lines (cubic polynomials) are for visualization purposes only. Approximate LSU regions for type-1 and type-2 networks are indicated (**a**, orange). We present also the edge length frequency (*f*) distributions (**c**,**f**), inter-edge (*θ*), dihedral (*φ*) and skew (*χ*) angle frequency distributions (**d**,**g**) and structure factor slices (**e**,**h**) for typical type-1 and type-2 networks, respectively. Structure factor intensities *I* are plotted on logarithmic colour scales and data in both panels is normalized to the maximum intensity *I*_0_ of **h**.

We investigated our ensemble of 216-vertex networks closely, calculating the LSU Φ_22_ distribution for each network. In Fig. 4a we plot the mean value 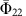 of each distribution against PBG width for all 57 networks. We see that gap width is strongly correlated with network LSU. We expect PBGs in amorphous gyroid to open within the gap region of a SNG PhC of equivalent index contrast. We thus defined the SNG gap as a critical frequency region, and counted the number of photonic bands that each of our 216-vertex networks support within this window. The number of bands within the critical frequency region can be interpreted as an integrated density of states (DOS). We plot the integrated DOS against 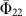 in Fig. 4b. We see that a network's inability to support electromagnetic modes is strongly correlated with its LSU (see also Supplementary Figs 2 and 3 and Supplementary Notes 1 and 2). Together these results demonstrate the power of LSU as a predictor of PBG forming ability.

We now discuss two characteristic types of amorphous gyroid in detail; we call these type-1 and type-2 networks. We characterize the networks through histograms of their geometrical properties. In particular, we measure edge lengths (*d*), inter-edge angles (*θ*), dihedral angles (*φ*) and skew angles (*χ*). Note that the skew angle measures the coplanarity of a trihedral unit, with *χ*=*π*/2 representing a flat trihedron (see Supplementary Methods).

Figure 4c,d show typical frequency distributions of *d*, *θ*, *φ* and *χ* for type-1 networks. They are characterized by strongly peaked edge-length and inter-edge angle distributions, but have non-uniform dihedral and skew angles. Type-1 networks can thus be considered high-quality amorphous networks of trihedral 1-tree units. Figure 4f,g show the same distributions for a typical type-2 network. Its *d*, *θ*, *φ* and *χ* distributions are all peaked around the ideal values for gyroidal vertices. Compared with type-1 networks, the local geometries of type-2 structures are much closer to an ideal strongly isotropic configuration. They possess gyroidal structural order on the length-scale of a 2-tree unit—the fundamental building block of SNG (Fig. 1d). Type-1 networks have 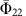 values around 0.72. Type-2 networks have significantly higher LSUs with 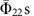 around 0.89. Typical Φ_22_ distributions for these networks are shown in Fig. 3d.

Figure 4e,h show planar slices through the structure factors of type-1 and type-2 networks, respectively. The structure factor is a quantity that characterizes a structure's coherent scattering power as a function of wavevector. We see that the peak scattering power of both network types is distributed in a circular ring on account of the average isotropy of the structures; wavevectors that lie on this ring are strongly scattered. We averaged the full structure factor across all propagation directions and measured the peak scattering power of type-2 networks to be approximately 1.6 times greater than type-1. In spite of this small increase, type-2 networks take the PBG width from practically zero to a maximum of 21%. We attribute these radically different PBG widths to the formation of locally self-uniform trees within type-2 networks.

We now demonstrate the natural connection between LSU and PBG formation in connected networks. In addition to the coherent scattering (Bragg) mechanism, there exists a Mie scattering mechanism^5^,^32^ of PBG formation. The Mie mechanism is known to be the dominant formation process for transverse magnetic (TM) polarization gaps in dielectric cylinder arrays. Specifically, sizeable PBGs exist in periodic^10^, quasicrystalline^18^ and random^6^ cylinder arrangements and are observed to be of the same origin in each case^32^.

The gap originates from resonant scattering by the Mie modes of a single cylinder. For all TM-gap cylinder arrays, it is clear from the electric field profiles at the edges of the fundamental PBG that Mie scattering mediates light propagation^6^,^10^,^18^. Just below the gap, modes are characterized by localization of field nodes in the vicinity of the cylinder surfaces. Just above the gap, field nodes consistently bisect the dielectric cylinders. These two node profiles derive from the interaction of a plane wave with an isolated cylinder, and are associated with the first and second Mie resonances, respectively^32^. Just above the first Mie resonance, incident and scattered fields are in antiphase at the cylinder surface; this creates a localized standing wavefront which inhibits propagation and leads to PBG formation^32^.

Existing evidence suggests that PBG formation in two-dimensional and 3D dielectric networks is governed by a similar mechanism of resonant scattering. Specifically, careful examination of the gap-edge eigenmodes of all honeycomb-derived networks presents a consistent picture of the nature of these scattering resonances. In both crystalline and hyperuniform disordered honeycombs^6^,^18^, modes just below the fundamental PBG are characterized magnetic field nodes localized within the dielectric network (conversely, field anti-nodes focus within the air cells). Just above the PBG, magnetic field nodes pass between air cells, cutting the inter-vertex dielectric walls almost normally. These gap-edge node characteristics are consistent across honeycomb-derived trivalent networks and, by analogy to the TM case, evidence the significance of spatially localized resonant scattering processes.

We therefore view CRNs as a connected ensemble of distinct scattering units (*n*-trees) which, in isolation, exhibit a number of resonant electromagnetic modes. For frequencies just above a resonance, scattered and incident fields are in antiphase and interfere destructively, localizing a field node in the vicinity of the scatterer and suppressing propagation through it.

A potential champion PBG structure comprises geometrically identical scattering units. All scattering centres thus possess degenerate electromagnetic resonances, and the spectral ranges for which each scatterer inhibits transmission are maximally aligned. Fixed valence networks comprising non-identical scattering units exhibit smaller PBGs than their crystalline precursors. Structural deformation of the scattering centres breaks the degeneracy of their scattering resonances; the resulting PBG is thus narrowed by imperfect overlap of the spectral ranges for which each scattering centre suppresses transmission.

The set of networks comprising geometrically identical scattering units exhibits a clear hierarchy of PBG size^33^ (see Supplementary Table 1). Specifically, networks built from vertices with a low coordination number possess the largest PBGs. Accordingly, the diamond and SNG architectures are champion and near-champion, respectively; these networks are strongly isotropic due to the simplicity of their vertices and comprise scattering units which are perfectly superimposable under permutation. This combinatorial symmetry has a strong influence on the PBG width. We argue that symmetry under permutation minimizes the number of distinct scattering resonances that a scattering centre supports. As a result, the frequency gaps between scattering resonances are maximized, together with the width of the spectral region above resonance for which transmission is suppressed.

Fundamentally, both Bragg and resonant scattering mechanisms contribute to PBG formation. The largest PBGs are obtained by optimization of a structure's dielectric fill fraction to overlap the spectral range associated with the two mechanisms. We note, however, that strong diffraction rings in the structure factor of amorphous materials do not directly lead to PBGs, but reflect the presence of local order that, depending on its LSU, may favour gap formation. This observation clarifies the relationship between LSU and work on PBG formation in hyperuniform structures^6^. Architectures derived from disordered hyperuniform point patterns possess significant local structural correlations and local geometrical order; these characteristics have proven essential in establishing sizeable PBGs^6^. However, hyperuniform point patterns must be tessellated in an *ad-hoc* way to produce viable PBG-forming networks^6^,^34^. This tessellation protocol naturally creates nearly-optimal network topologies, but these networks are successful only because they possess locally self-uniform structural order. In contrast to hyperuniformity, LSU measures both geometrical and topological order simultaneously and is thus an effective measure of PBG forming ability. Hyperuniformity and LSU remain compatible; we note the emergence of a hyperuniform-like exclusion domain around ***k***=0 in the structure factor of networks with significant LSU (Fig. 4h). The association of LSU with PBG formation parallels the proof that amorphous materials with well-defined atomic connectivity can possess an electronic bandgap^35^.

### Microwave experiments with amorphous gyroid

**Figure 5:**
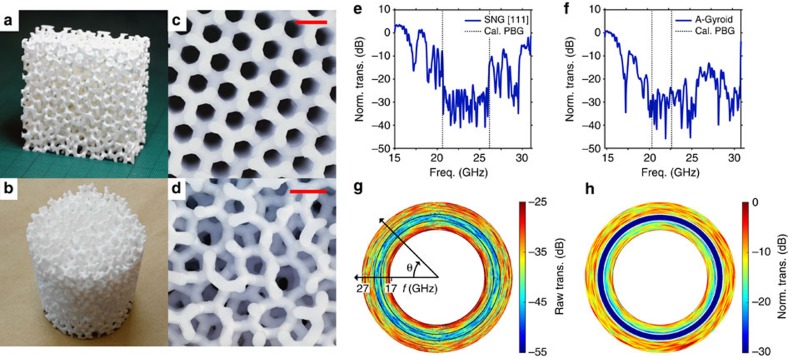
Microwave experiments with single network and amorphous gyroid structures. Alumina prototypes of amorphous gyroid, both a single piece cuboid (**a**) and a compound cylinder (**b**). View down the (111) axis of our single-network gyroid sample (**c**), and along an arbitrary axis of amorphous gyroid (**d**), showing the network quality. Comparison of transmission between single-network gyroid (SNG [111]) and amorphous gyroid (A-Gyroid) cuboidal samples (**e**,**f**, respectively), with gap edges predicted by plane wave expansion (vertical dashed lines); measured data was smoothed by Fourier filtering for clarity. Measured (**g**) and simulated (**h**) polar false-colour maps of transmission for the amorphous gyroid cylinder, with gap edges calculated by band structure overlaid as black and white rings, respectively. Scale bars in (**c**,**d**) 5 mm.

To verify our theoretical calculations, we fabricated millimetre-scale amorphous gyroid samples and experimentally characterized their PBGs. Samples were produced at the Fraunhofer Institute for Ceramic Technologies and Systems using a 3D ceramic printing technique. The samples were made from alumina (Al_2_O_3_), whose permittivity was experimentally determined to be 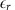
=(9.5±0.3) at frequencies in the microwave *K* band (18–26.5 GHz). Two types of sample were made: cuboidal samples of SNG (Fig. 5c) and amorphous gyroid (Fig. 5a,d), and a cylindrical sample of amorphous gyroid (Fig. 5b). The internal network, comprising cylinders with diameter *D*=2.03 mm, was well formed (Fig. 5c,d). The gyroid primitive cell parameter was designed to be 3.08 mm and experimentally measured to be *a*=(3.13±0.05) mm.

Measurements were made with microwave radiation using an established experimental set-up^9^,^19^. Each sample was placed between two facing microwave horn antennae connected to an HP-8510C vector network analyser (VNA). Figure 5e,f present the transmission as a function of frequency along the (111) crystal axis of SNG and an arbitrary amorphous gyroid axis, respectively. Both samples show wide transmission gaps, with up to 35 dB of attenuation. Propagation through the periodic sample (Fig. 5e,f) is predominantly ballistic^9^; photons are weakly scattered both above and below the PBG, and the gap edges are well defined. As a result, the measured transmission gap for the SNG sample agrees very well with the simulated PBG, highlighted by vertical dashed lines in the figure.

The measured transmission gap for the amorphous gyroid sample (Fig. 5f) is also centred at the frequency range calculated by simulation (shown as dashed lines), although it appears wider; we attribute this to the limited dynamic range of our experiment (see Methods section) and strong diffusive scattering^9^. Scattering processes are significant for the amorphous network. They scatter radiation both away from the detector and into different polarization states. As a result, strongly scattered radiation has a low coupling efficiency into the receiver horn antenna; this prevents the transmission from recovering its peak value for frequencies above the PBG, thus widening the perceived transmission gap.

We employed the amorphous gyroid cylindrical sample (Fig. 5b) to investigate the isotropy of the PBG. The sample was rotated around its cylindrical axis and a transmission spectrum was recorded every 2°. The resulting set of transmission spectra is presented in Fig. 5g as a polar, false-color map in which the radial coordinate represents frequency and the angular coordinate records the cylinder's rotation angle^36^,^37^. Here, the rotational isotropy of the PBG is clear; the blue-and-green ring represents an isotropic transmission gap. The expected PBG edges, as predicted by band structure calculation, are overlaid as black solid curves. We corroborate the PBG isotropy by performing finite-difference time-domain (FDTD) electromagnetic simulations. Specifically, we place a number of dipole sources inside our amorphous gyroid cylinder, and record the power flux some distance from the cylinder using a circular array of detectors (see Supplementary Methods). This result is presented as a second polar false-colour map in Fig. 5h; the expected PBG edges, according to the band structure, are overlaid as white solid curves. Although the transmission contrast of the experimental data is noise-limited (see Supplementary Note 3), the frequencies of the experimentally measured PBGs accord well with the results of both band structure and FDTD simulations.

### Self-uniformity by evolution

Many plants and animals have evolved wavelength-scale microstructures as means of producing colour^38^,^39^,^40^,^41^. Study of these architectures can inspire the development of industrial scale fabrication techniques for next-generation PhC-based technologies^42^,^43^,^44^.

In this regard, self-assembly is a particularly attractive fabrication method. The three strongly isotropic networks have all been observed to self-assemble^45^,^46^,^47^,^48^. Interestingly, amorphous honeycomb and diamond have also been observed in the natural world^49^,^50^; it is thus clear that a self-assembly pathway capable of producing complex short-range order exists. Here, we explore evidence that an amorphous gyroid could be similarly self-assembled. First, we demonstrate that topological defects exist in the gyroidal microstructures of green hairstreak butterflies^47^,^51^. We then present a disordered network structure in the scales of the Cambridge Blue butterfly and model its reflectance spectrum with an amorphous gyroid structure^52^.

Many butterfly species are known to derive colouration from gyroidal networks of chitin within their scales, most famously *Parides sesostris* (the emerald-patched cattleheart) and numerous species of the genus *Callophrys* (the green hairstreaks). Microscopy has demonstrated that the scales contain numerous crystallites of a well-ordered network of chitin in air^51^,^53^. Small angle X-ray scattering (SAXS) has assigned the symmetry group of SNG to the structures^47^.

However, tomographic reconstruction of the chitin/air interface in *Callophrys rubi* shows that the network is not everywhere a perfect topological gyroid^51^. Evidence suggests that the chitin network passes continuously between adjacent gyroid grains, and that this grain-matching is facilitated by topological network defects^51^. Further, different crystallites within the same scale exhibit different chitin fill fractions. Accounting for these observations, we now show that SNG crystallites incorporating small amounts of topological and positional disorder are better models of the scale structure in the green hairstreaks than a perfect gyroid crystal.

**Figure 6:**
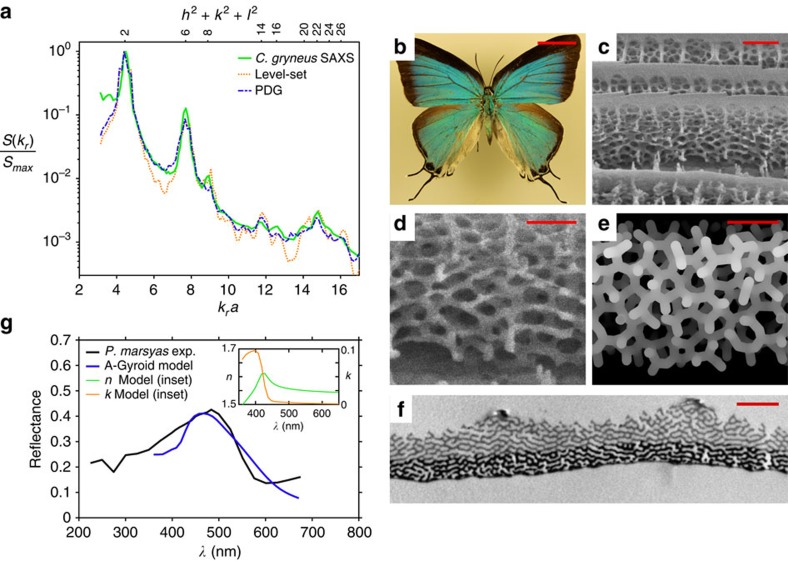
LSU in butterfly scales. Comparison with SAXS data of level-set and partially disordered gyroid models of wing scales in the butterfly *C. gryneus* (**a**). Specimen of *Ps. marsyas* (**b**) and FIB section of one of its blue scales (**c**), imaged with 52° tilt in the FIB. Detail (**d**), taken from (**c**), compared with a projection of an amorphous gyroid model (**e**). Transmission electron micrograph of a *Ps. marsyas* scale cross-section (**f**). Comparison of experimental reflectance data from a large wing area to an amorphous gyroid scale model (**g**). Scale bars, 1 cm (**b**); 1 μm (**c**); 500 nm (**d**,**e**); and 1.5 μm (**f**). The TEM section (**f**) is reproduced with permission from Vértesy *et al*.^52^. TEM, transmission electron microscopy.

To model the SAXS results, we approximate the scale as an ensemble of separate crystallites which contribute to the diffraction spectrum independently. The positional correlations between adjacent crystal domains are destroyed by the matching defects at the grain boundaries; the SAXS spectrum can thus be considered an incoherent superposition of the diffraction patterns of distinct crystallites. To fit the experimental results, we generate a number of distinct crystallites across a range of chitin fill fractions and sum their diffraction patterns incoherently, optimizing their weights in the summation to minimize the Reitveld weighted profile *R* factor, *R*_*wp*_, of the fit^54^. We calculate two types of fits, described in detail in the Methods section. In the first case, we model the scales as perfect gyroids, employing the level-set approximation of the gyroid minimal surface (dotted orange curve in Fig. 6a). In the second case, we model the tomographic data^51^ using partially disordered gyroid (PDG) networks (dashed blue line in Fig. 6a). PDG is generated by introducing a small number of topological defects into a perfectly ordered gyroid net. Specifically, one defect is introduced for every 100 network vertices together with a small amount of vertex positional disorder.

Note that, at publication, no SAXS data for *C. rubi* was available. Instead we compare our models to SAXS results for *Callophrys gryneus* (solid green line in Fig. 6a); we expect the microstructure in *C. gryneus* to comprise gyroid grains connected by topological matching defects, just as in *C. rubi*. *R*_*wp*_ values for our level-set and PDG fits are 980 and 645, respectively, suggesting that PDG is a superior model of the green hairstreak scale structure. The pure level-set model produces overly prominent peaks, particularly between the (110) and (211), and (400) and (420) reflections. The inclusion of topological and positional disorder dampens the network correlations and reduces this contrast across the whole spectrum. The quality of the disordered PDG fit is most evident for high (*hkl*) values; here, the overall profile of the pattern, in particular the double peak of the (321) and (400) planes, is well-captured.

Taken together, the tomographic data^51^ and the results of our scale modelling (Fig. 6a) suggest that topological imperfections form, to a limited degree, at the grain boundaries in the gyroidal microstructures of the green hairstreaks. The existence of these topological defects renders the hairstreaks (sub-family Theclinae) a promising family of butterflies within which to search for an amorphous gyroid.

We now turn our attention to the Cambridge Blue butterfly (*Ps. marsyas*, Theclinae, Fig. 6b). Its wing scales contain a unique aperiodic photonic structuring from which they derive a blue brilliance. Focussed ion beam (FIB) sections reveal an intricately connected trivalent network, with fully 3D interconnectivity and an apparent LSU (Fig. 6c). Computational projections of amorphous gyroid with a volume fill fraction of 25% strongly resemble the butterfly network (Fig. 6d,e). Transmission electron microscopy sections (Fig. 6f) reveal a mixture of interwoven layering and fully disordered network regions^52^. In cross-section, the lower half of the scale (bottom half of Fig. 6f) appears as a dark band—this increased contrast can be associated with pigmentation^55^.

We now model scales of *Ps. marsyas* as amorphous gyroid CRNs and investigate the compatibility of these models with experimental reflectance measurements. We employ a number of amorphous gyroid CRNs with 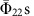 around 0.88. We calculate their far field reflectance spectra by FDTD solution of the Maxwell equations.

We estimated the scale thickness from FIB sections to be 1300±200 nm. The lower half of the scale was taken to be absorbing in accordance with the pigment distribution observed by transmission electron microscopy (Fig. 6f). We suggest a plausible model for the complex refractive index of the *Ps. marsyas* pigment, derived from the extinction coefficient of the pigment in *Papilio nireus* (see the Methods section). Amorphous gyroid edge lengths for our *Ps. marsyas* sample were estimated from electron micrographs to be 117±6 nm; this corresponds to an effective SNG primitive cell parameter of *a*=166±8 nm. The reflectance of a large wing area of *Ps. marsyas* was measured by a previous study^52^; scaling of the amorphous gyroid (110)-type SAXS peak to the wavelength of maximum reflectance suggests *a*=169 nm. The theoretical reflectance presented (Fig. 6g) is an average over six amorphous gyroid models, all scaled by an *a* value of 166 nm.

Given the assumptions made in modelling the pigmentation, the general agreement between theoretical and experimental reflectance spectra suggests that an amorphous gyroid with a UV-absorbing pigment is a plausible model of the structure in *Ps. marsyas* scales. The divergence at UV wavelengths is attributable to uncertainty in the exact complex refractive index of the butterfly scale across a 300 to 700 nm range; future studies should measure this directly. The small divergence at red wavelengths is attributable to reflections from unmodelled melanized ground scales.

Two other neotropical hairstreaks, *Arcas imperialis* (Theclinae) and *Evenus coronata* (Theclinae)—close relatives of the Cambridge Blue—have previously been surveyed through microscopy^56^. Their structures appear to possess strong multilayer and gyroidal characteristics, respectively. It is therefore possible that amorphous gyroid has evolved in the Cambridge Blue by some small change in scale cell development conditions, leading to an evolutionary divergence. Indeed, such a divergence has been postulated in the case of the gyroid-containing *Pa sesostris*, whose-scale structuring has diverged from the perforated multilayers of many closely related *Parides* species^57^.

Our work indicates that the scale structuring in the Cambridge Blue is related to an amorphous gyroid. Electromagnetic modelling shows that amorphous gyroid models are consistent with observed reflectance data and the existence of topological matching defects between gyroid grains in green hairstreak scales suggests that the production of amorphous gyroid is developmentally possible.

Any further search for a natural amorphous gyroid should not be limited to butterflies. A thorough survey of avian feather barbs has revealed an abundance of colour-producing channel-type architectures. Several species—*Diglossa cyanae*^58^ (Thraupidae), *Passerina cyanea*^58^ (Cardinalidae) and *Alcedo atthis*^59^ (Alcedinidae)—are themselves a brilliant blue and possess microstructures with striking similarity to amorphous gyroid. Experimentally, it may be possible to produce amorphous gyroid or a similar architecture via block co-polymer self-assembly^60^. It is possible to generate mixed lamellar and gyroidal states that may resemble the *Ps. marsyas* structure^61^,^62^. An alternative pathway may be to quench the phase transition between the gyroid and metastable perforated multilayer phases^62^.

## Discussion

We have introduced LSU as means of measuring the extent to which local environments in a CRN of fixed valency are spatially similar. When applied to strictly trivalent and tetravalent networks the states of maximum LSU are the strongly isotropic single gyroid and diamond networks, respectively. As a network's LSU decreases from its maximum it becomes glassy and eventually chaotic. LSU can thus classify all CRNs of fixed valency by the extent of their internal order.

We designed LSU as a means of characterizing the optical properties of CRNs. In particular, we have shown that networks with maximal LSU possess champion PBGs in both 2D and 3D. Further, other known networks endowed with a complete PBG in 3D or a TE polarization PBG in 2D are characterized by high levels of LSU. PBGs result from scattering by the electromagnetic resonances of a network's local scattering units. When these units are spatially similar, their resonances are maximally degenerate and complete PBG formation is favoured. LSU is thus a predictive measure of a network's PBG forming ability. While here we focussed on trivalent and tetravalent networks, LSU can be generalized to include networks of arbitrary or mixed valency.

We have introduced designs of novel amorphous gyroid CRNs. Here we used ceramic 3D printing to fabricate amorphous gyroid samples in high-refractive index alumina and demonstrated their sizeable isotropic PBGs via microwave transmission experiments. The relevance of amorphous gyroid, and architectures which can be derived from it, is broad. In particular, its development, or that of a closely related CRN, appears to have occurred in the scales of the butterfly *Ps. marsyas*. That it is possible to self-assemble such a structure is a prerequisite for its existence in natural systems. It may be possible to produce amorphous gyroid networks via block co-polymer experiments or equivalent self-assembly methods. This poses an interesting experimental challenge, the solution of which will facilitate the fabrication of advanced optical metamaterials for industrial applications^43^,^60^.

Fundamentally, we have demonstrated that the tree comparison method is a powerful framework for controlling the LSU of the scattering centres in a CRN. Sculpting a network's LSU distributions translates directly to advanced control over its optical properties. The optical properties controlled need not be limited to PBG forming ability; they could include structural colour, the scattering mean free path^63^ and random lasing^64^. Moreover, similar design principles may be employed to control other wave phenomena in electronic, phononic, elastic and acoustic materials.

## Methods

### Definition of the LSU distributions

Consider a CRN *C* with a set of defined vertex positions and inter-vertex connectivities, in which each vertex in *C* has exactly *γ* edges. We define an *n*-tree 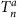 on vertex *a* of *C* as the set of vertices within *n* edges of *a*. The root edges of 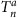 are all the edges of 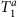. Computationally, all information regarding 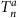 can be obtained by performing a breadth-first graph search to depth *n* from vertex *a*.

We consider now two *n*-trees of equal depth 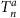 and 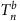. We label the root edges of the trees {1_*a*_, 2_*a*_…*γ*_*a*_} and {1_*b*_, 2_*b*_ ... *γ*_*b*_}, respectively. The spatial similarity statistic *φ*_*ab*_ of these two trees is defined as


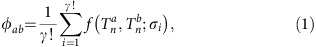


where *f* is a similarity measure which grades the overlap of 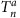 and 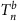 when they are maximally aligned in a root edge permutation *σ*_*i*_. We sum the similarities for all *γ*! overlap permutations of the trees' root edges and then take the average.

The value of the spatial similarity statistic will depend both on the form that the measure *f* takes and the method that is used to maximally align the two trees for each permutation. We describe our choice of *f* and the alignment procedure we follow in the next section.

The LSU distributions of the CRN are particular sets of spatial similarity statistics. We define a new tree 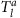 with depth *l* on vertex *a*. The set of vertices in 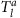 is the local neighbourhood of *a* to depth *l*. The LSU distribution Φ_*nl*_ can then be written as


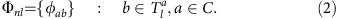


Φ_*nl*_ is thus the set of spatial similarity statistics for all trees of depth *n* whose root vertices are within *l* edges of one another. Φ_*nl*_ can be plotted as histograms as in Fig. 3. In Fig. 3 the histogram frequencies are normalized through division by the total number of spatial similarity statistics in Φ_*nl*_.

### Calculation of the overlap between two trees

Here we discuss in detail the exact form of our similarity measure *f* and the process by which two trees are maximally overlapped. We note that the choice of *f* is an important user-controllable degree of freedom. In general, *f* must be maximal when two trees can be overlapped perfectly; this defines uniquely the maximally self-uniform configuration. However, the exact manner in which overlap is calculated can be defined to best suit the application and to possess meaningful properties as the network departs from maximal self-uniformity. Here, we adopt an intuitive framework in which tree edges are overlapped in pairs with their most natural partners.

Consider now an *n*-tree 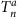 in the CRN *C*. We label its 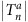 vertices with index *j*, subject to the constraint that indices {1, 2 ... *γ*} represent the tree's root edges. The branches of the tree are defined by its edge vectors. For 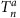 we denote these as 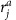, which defines the vector to vertex *j* from its parent vertex as defined by a breadth-first graph search starting on *a*.

Consider now two *n*-trees 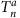 and 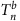. Before they can be compared, they must be maximally overlapped. This process consists of determining a congruent transformation which maximizes the overlap of their root edges in some chosen permutation *σ*_*i*_. First, 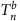 is translated such that vertices *a* and *b* overlap. Second, 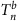 is rotated until 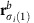 and 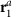 are parallel; this is always possible. Third, 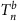 is rotated about the 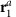 axis until 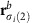 and 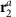 are maximally parallel, defined to be the configuration which minimizes their scalar product. For disordered trees, it is not usually possible to make these two vectors perfectly parallel. For trivalent networks, we deem these three steps sufficient to maximally align the two trees.

For tetravalent networks it is necessary to introduce a fourth alignment step. In this case, 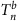 is reflected in the plane defined by 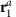 and 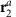 so as to bring 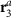 into maximal alignment with 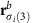 and 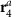 with 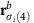. This step is performed only if the alignment between 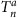 and 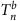 is improved, as measured by an increase in the value of 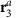
·
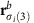
+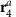
·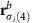. At this point, the two trees are considered to be maximally aligned.

Once maximally aligned, we define our similarity measure *f* for grading the overlap of two trees as


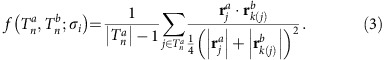


Overlap is calculated between edge pairs by taking their scalar product and normalizing it with the square of their mean norm; overlap of a single pairing is thus distributed on [−1, 1]. The 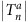
−1 edge pair comparisons are summed and averaged such that *f* yields a maximum value of unity for perfectly overlapping trees.

Edge pairs are overlapped in the combination that is most natural. The whole overlap calculation is performed recursively in a depth-first sense, greatly simplifying the process of determining natural pairings. At a particular point in the algorithm's execution, it has reached some vertex pair *j* and *k*(*j*) by comparison of the natural edge pair 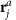 with 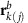. Overlap of all (*γ*−1)! possible sets of edge pair comparisons is calculated. The set that maximizes the sum of edge pair scalar products is chosen as the set of natural pairings; this result is accepted, and the algorithm proceeds to calculate overlap for the edges around each of the (*γ*−1) child vertices. This decision process is captured in the notation 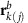, to reflect that the choice of edge vector in tree *b* is a function of the edge vector 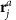 to which it is being compared.

Finally, the spatial similarity statistic *φ*_*ab*_ is determined by repeating this process for all root edge permutations according to equation (1).

### Microwave transmission measurement

Gyroid and amorphous gyroid models were printed and finished in the workshops of the Fraunhofer Institute for Ceramic Technologies and Systems, Dresden. The gyroid primitive cell parameter was designed to be to be 3.08 mm. As part of the printing process, the samples were sintered at high temperature, shrinking in side-length by ∼20% and introducing some uncertainty into their true sizes. Actual scaling values were thus measured and fill fractions were determined using a water displacement method, yielding an effective gyroid primitive cell parameter of *a*=3.13±0.05 mm, and alumina volume-fill-fractions between 27 and 29%. Amorphous gyroid samples were fabricated preferentially. We also produced one SNG model. This model was designed for measurement of transmission along the [111] axis; this axis was chosen because of its symmetry (see Supplementary Note 4).

Characterization of the samples was performed with microwave radiation using an HP-8510C VNA. A single polarization mode was coupled through a pair of rectangular horn antennae and a pair of custom-made Teflon microwave lenses. For the measurements shown in Fig. 5e,f, the beam was aligned along the short edge of the cuboidal samples (Fig. 5a,c) and perpendicular to a flat sample surface, and the frequency varied from 15 to 35 GHz. Note that frequencies beyond the standard microwave K band (18–26.5 GHz) have very low efficiency coupling through the horns and waveguides. For the measurements shown in Fig. 5g, the cylindrical sample was aligned with the incident beam perpendicular to the cylinder axis, around which we rotated the sample and recorded the transmission every 2°. This set of measurements was performed across a frequency range of 17–27 GHz.

The total dynamic range of the HP-8510C VNA is from 0 to −65 dB (measured dark noise). A large area of microwave-absorbing material, into which a window was cut to hold the sample, was used to prevent reflection and scattering of radiation into the environment. The normalized transmission is then defined as the ratio between detected intensities with and without the sample in place. The addition of microwave-absorbing material lowers the overall coupling-efficiency through the pair of horn-antennae. As a result, the actual accessible dynamic range through this measurement is only ∼35 dB, which limits the measured gap-depth. This limited dynamic range is apparent in the amorphous gyroid transmission of Fig. 5f; the transmission bottoms out and becomes noisy for frequencies between 20 and 26 GHz. For the same reason, transmission results for the amorphous gyroid cylinder (Fig. 5g) appear noisy in comparison to the theoretical results (Fig. 5h). An increased dynamic range could be accessed by amplifying the source power.

### SAXS pattern modelling

The total scattered intensity measured in a SAXS experiment can be written as a Fourier transform of the electron density function *ρ*(**r**):


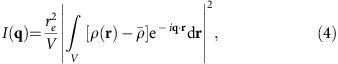


for **q** the scattering vector in reciprocal space, *r*_*e*_ the radius of the electron and *V* the total sample volume^65^. We Fourier transform the difference between *ρ*(**r**) and its volume average 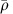 to remove the forward scattering peak and access any diffraction signatures that may be present at small scattering vectors. The *ρ*(**r**) functions for our 3D network structures were generated by voxelization and were distributed on [0, 1], representing the air and chitin distributions of the butterfly scale networks, respectively. Fourier transforms were calculated using a fast Fourier transform algorithm. Powder-like structure factors were calculated by azimuthal averaging of the total scattered intensity. Structure factors were normalized to the intensity of the (110) peak for comparison with experimental data.

To model the observed scattering from *C*. *gryneus*, we investigated both partially disordered gyroid (PDG) networks and level-set surfaces as approximations to the chitin network. In all cases, cubic crystallites of dimension 10*a* (1,000 vertex points) were used. When *f*=0, the function


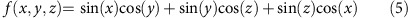


defines the level-set approximation to the gyroid surface; it divides space into two inter-penetrating labyrinths each with a 50% volume fill. For *f≠*0, it defines a surface with the symmetry of SNG, and alters the relative filling fraction of the two labyrinths. A set of level-set basis states was generated by sampling these different fill fractions via a critical value *t*, assigning unity to *ρ*(**r**) for *f*>*t* and zero otherwise.

PDG networks were generated by introducing geometrical and topological disorder into SNGs. All PDG networks were topologically disordered by the introduction of 10 Stone–Wales defects. This was performed using our implementation of the WWW algorithm. Geometrical disorder was added by perturbation of the vertex points by a random vector whose magnitude was normally distributed with mean zero and s.d. *σ* up to 40% of the gyroid edge length. To increase the physicality of this disorder, perturbations were propagated through the network with their magnitude decaying with depth; vertex positions thus retained a local correlation. The final set of disordered SNG basis states sampled various degrees of vertex positional disorder, as defined by their perturbation standard deviations and rates of perturbation decay, each across a set of fill fractions.

Final diffraction spectra consist of a weighted sum over the diffraction patterns of the appropriate basis states. A fit to the experimental data was performed by optimization of these weights so as to minimize the Rietveld weighted profile factor of the fit.

### *Pseudolycaena marsyas* scale imaging

A dried male specimen of *Ps. marsyas* was acquired online from The Bugmaniac Insect Shop. The specimen was spread and mounted for sample photography. Single scales were removed, mounted and gold sputtered to produce a 4 nm coating. Scales were then imaged via electron microscopy, and sectioned using a FIB.

SNG net scaling parameters were estimated from micrographs under the assumption that network regions with a locally honeycomb-like appearance correspond to the nine-segment helices visible along the SNG (111) axis. The appearance of such regions is similar to views along the (111) axis of gyroid crystallites in green hairstreak butterflies, supporting this supposition. The radius *r* of such a helix satisfies the approximate relation 2*πr*=7.732*l*, for *l* the SNG edge length.

### *Pseudolycaena marsyas* reflectance modelling

Models of *Ps. marsyas* scales were generated using type-2 amorphous gyroid networks with 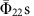 around 0.88. The effective single-network gyroid primitive unit cell dimension was estimated from micrographs of *Ps. marsyas* to be *a*=166±8 nm, corresponding to an edge length of *l*=117±6 nm. Approximating the scale's reflectance as a result of coherent scattering from (111)-plane-type reflections suggests *a*=169 nm.

Six models were made, each of thickness 1,350 nm and scaled with an *a* value of 166 nm. To model the apparent variation in cross-member thickness within the scale's network, amorphous gyroid point patterns were decorated with cylinders having normally distributed radii *r*, with a mean fractional radius *r*/*l*=0.44 and a 15% s.d. The resulting network has a mean volume fill fraction of ∼25%.

The Maxwell equations were solved using the Lumerical's implementation of the FDTD method (Lumerical Solutions, Inc). The previously published experimental data was gathered through normal illumination of a large wing area and use of an integrating sphere in reflection. To model this, scales were illuminated from above at normal incidence with a total field scattered field source. The scattered fields were recorded by a monitor box, and were projected into the far field in the upper half-space, back along the axis of incidence. The total power scattered into the upper half-space was determined and normalized to the injected power. Reflectance data was calculated across the wavelength range 360–670 nm using a uniform 11.5 nm mesh.

Cylinders in the upper half of each scale were modelled as a dispersive material with the refractive index of chitin as measured by polarizing interference microscopy applied to the butterfly *Graphium sarpedon*^66^. In accordance with an existing study^55^, the dark banding of the lower half of the *Ps. marsyas* scale was interpreted as pigmentation. The absorbance of this pigmentation was not measured experimentally. Rather, we suggest that the pigment is ultraviolet absorbing, as has been shown to be the case for *Papilio nireus*^55^, the gyroid-containing butterfly *Pa. sesostris*^53^ and many other structurally-coloured butterflies^67^.

To make progress in modelling the reflectance of *Ps. marsyas* scales, we propose a plausible complex refractive index for the pigmented lower layer. We derive the extinction coefficient *κ*(*ω*) of the ultraviolet-absorbing pigment in *Papilio nireus* using the Beer–Lambert law^55^. The real component of the refractive index was taken to be equal to that of the upper layer of the scale. Lumerical's in-built refractive index fitting algorithm was used to fit this combination of *n* and *κ*; extra weight was given to the imaginary component to accurately model the absorption. The resulting complex refractive index used for the lower half of the scales is inset into Fig. 6g.

The reflectance spectrum of Fig. 6g should be interpreted in light of the assumptions we have made in modelling the complex refractive index of the pigment. Given these assumptions, the agreement between the theoretical and experimental reflectance data shows that an amorphous gyroid network is a plausible model for the structure in *Ps. marsyas* scales. The divergence between theoretical and experimental reflectance at ultraviolet wavelengths is a result of uncertainty in the true refractive index of the *Ps. marsyas* pigment in this wavelength range. Further modelling of *Ps. marsyas* scales should be informed by a direct measurement of their complex refractive index^55^.

### Data availability

The data underlying the findings of this study are available without restriction. Details of the data and how to request access are available from the University of Surrey publications repository: http://dx.doi.org/10.15126/surreydata.00813094.

## Additional information

**How to cite this article:** Sellers, S. R. *et al*. Local self-uniformity in photonic networks. *Nat. Commun.* **8,** 14439 doi: 10.1038/ncomms14439 (2017).

**Publisher's note:** Springer Nature remains neutral with regard to jurisdictional claims in published maps and institutional affiliations.

## Supplementary Material

Supplementary Information Supplementary Figures, Supplementary Tables, Supplementary Notes, Supplementary Methods and Supplementary References.
