# Supplementary material for: Local self-uniformity in photonic networks
Source: Nat Commun. 2017 Feb 17;8:14439. doi: 10.1038/ncomms14439 (PMC5321726; doi:10.1038/ncomms14439)
Supplement: Supplementary Information — Supplementary Figures, Supplementary Tables, Supplementary Notes, Supplementary Methods and Supplementary References. [file ncomms14439-s1.pdf]

Supplementary Figures

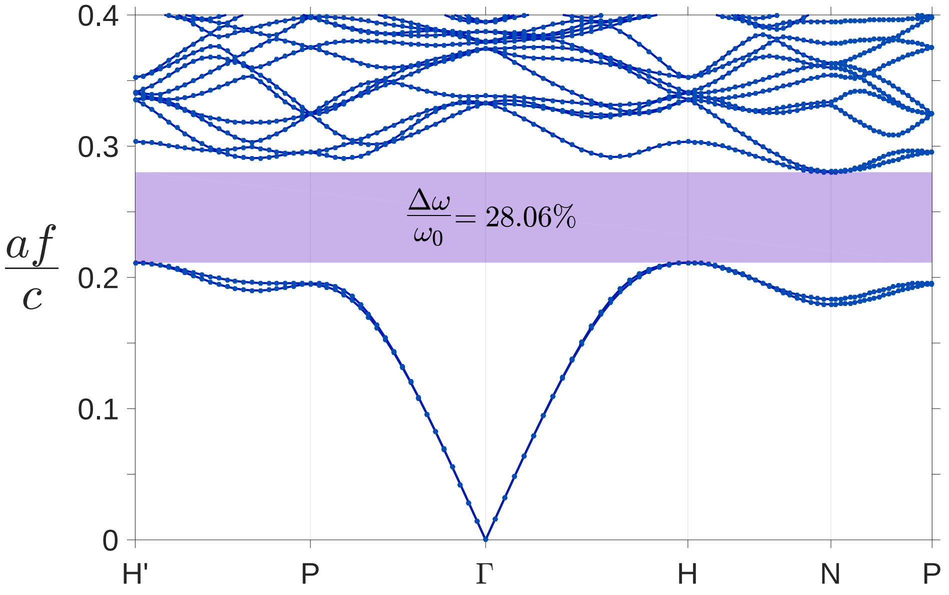

Supplementary Figure 1: **Photonic band structure for rod-decorated single network gyroid.** The photonic band structure is evaluated for an optimal rod-decorated single network gyroid photonic crystal comprising air and dielectric material of permittivity $\varepsilon_r = 13$. The radius of the dielectric cylinders was optimised to maximise the photonic band gap (PBG) between the second and third bands. The optimum structure comprises dielectric cylinders of radius $r/a = 0.2554$ where a is the single network gyroid primitive cell side length. The structure has a dielectric volume fill fraction of 17.88%. The PBG is located between bands 2 and 3 (shaded area) and has width 28.06%. This is simiilar to the 30% PBG in rod-decorated diamond[1] and renders the single network gyroid PBG near-champion.

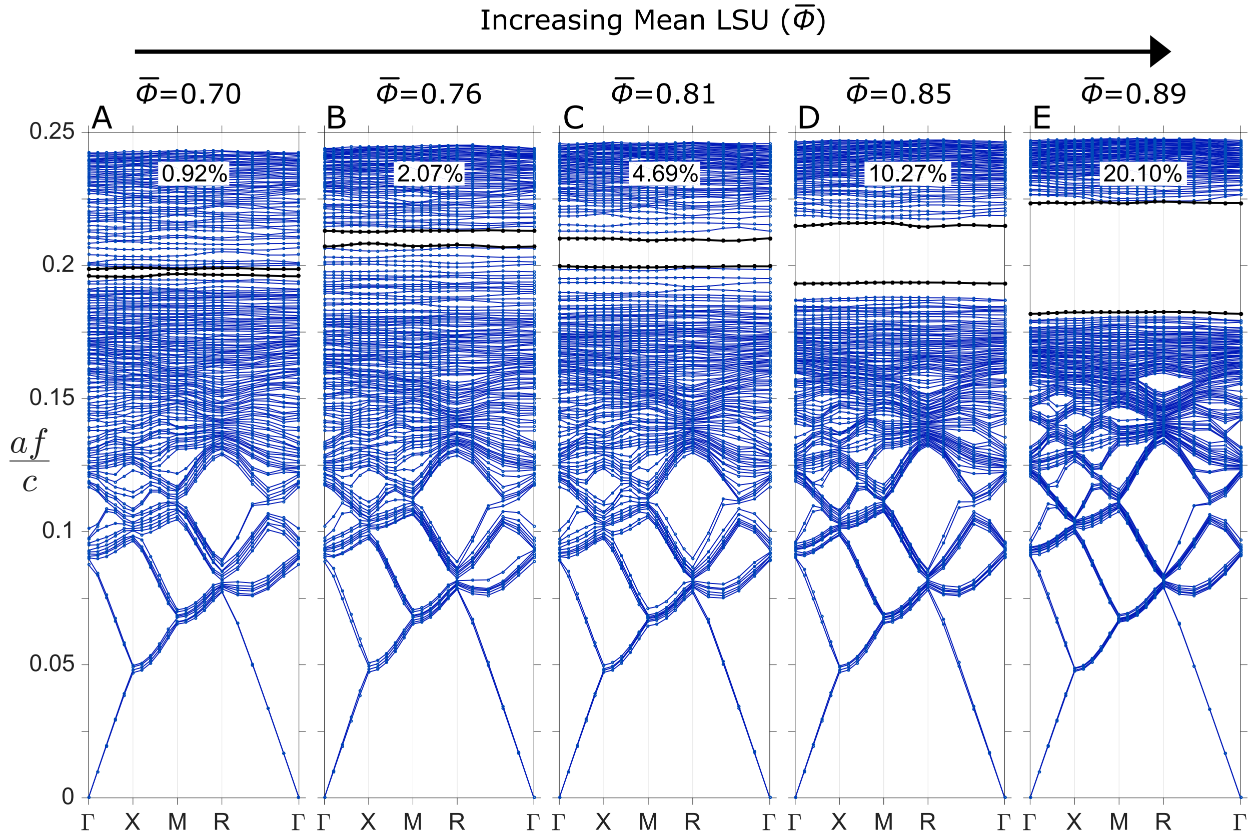

Supplementary Figure 2: **Evolution of photonic band structure with mean LSU.** Five band structures for 216-vertex amorphous gyroid models, showing the evolution of the band gaps with increasing local self-uniformity.

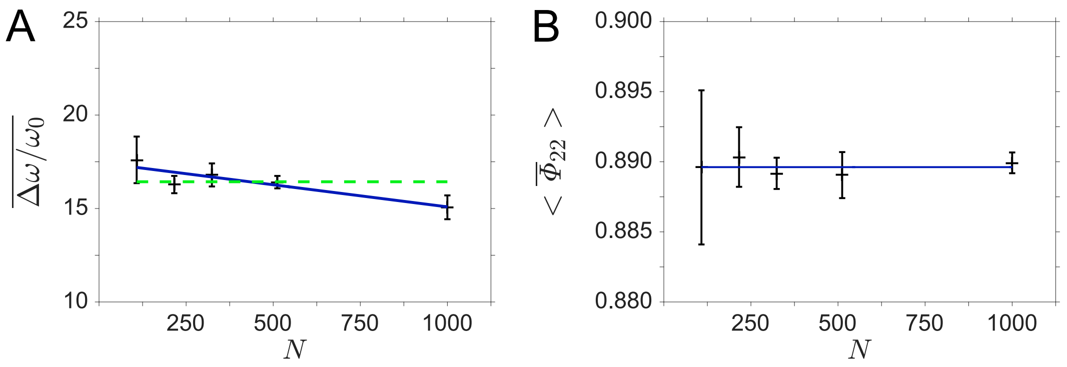

Supplementary Figure 3: **Evolution of the PBG width with network size**. We studied five ensembles of amorphous gyroid structures, where each ensemble was defined by the size of the amorphous gyroid networks contained therein. We calculated the mean PBG width for each ensemble and plotted this against network size (A); error bars describe the standard error on the mean. Each set comprised networks with similar $\overline{\Phi}_{22}$ values. The mean $\overline{\Phi}_{22}$ of each ensemble remains fairly constant (B); error bars show the standard deviation of the $\overline{\Phi}_{22}$ values within each ensemble. The blue, continuous line is a linear fit of the data and the dashed green line is a best constant-value fit.

Structure	Lattice	Gap Width	Co-ordination	Comments
Single Network Gyroid	BCC	28.1%	3	Near-champion
Diamond Network	FCC	31.4% *	4	Champion
Topological Diamonds	Various	Sizeable[1]	4	-
Amorphous Diamond	None	18%[2]	4	-
Inverse Opal	FCC	6%[3]	Mixed 4 & 8	Vertices at close-packed interstices
Opal	FCC	None[3]	Isolated spheres	-
Simple Cubic	PC	8.4% *	6	Larger gap when not rod-decorated[4]
FCC8	FCC	6.1% *	12	Larger gap when not rod-decorated[4]

Supplementary Table 1: **PBGs for various photonic-crystal architectures.** The major types of three-dimensional photonic crystal architectures and their corresponding PBG sizes at a permittivity contrast of $13 : 1$ when decorated with dielectric cylinders. The * labelled results are our own calculations for optimal rod-decorated networks.

Supplementary Note 1: PBG Size versus LSU

We note that connected networks favour PBG formation. All known complete (sizeable) PBG architectures in three-dimensions comprise connected dielectric networks[1,5,6]. Opal comprises isolated dielectric spheres in a cubic close-packed arrangement; it exemplifies a high-symmetry non-network structure which, in spite of its maximally-spherical Brillouin zone, exhibits no complete PBG.

Brillouin zone isotropy is a poor predictor of PBG size. Opal and inverse opal structures are both described by the fcc lattice, whose BZ is maximally spherical. The inverse opal gap has width 6% while the opal structure is gap-less. A rod-decorated simple cubic architecture has a PBG of width 8.4%[7], which is larger than inverse opal in spite of its less isotropic BZ; simple cubic can display a significantly larger gap of width 16.3% when not rod-decorated[4]. Similarly, numerous non-FCC topological diamonds[1] possess sizeable gaps in spite of the reduced isotropy of their BZs. Further amorphous diamond and gyroid networks possess no BZ and display significant gaps in spite of their non-Bragg-like structure factors.

Instead, the common property of champion and near-champion PBG networks is the low coordination number of their component network vertices; Suplemantary Table 1 shows that the PBG width reduces as the vertex coordination number increases from 3 to 12. Further, all but one of the sizeable PBG networks discussed are characterised by locally (and globally) uniform vertex connectivities. Non-uniform local connectivity, like that of the inverse opal network, appears to disfavour gap formation. Given a particular vertex coordination number, the largest PBGs occur in networks for which the local vertex configurations are highly symmetric. As the local vertex symmetry is broken, and a network becomes increasingly disordered, its PBG size appears to decrease. Significantly, the champion and near-champion diamond and single network gyroid structures are both strongly isotropic as a result of the simplicity of their component vertices[8].

Overall, we synthesise these observations into our measure of local self-uniformity (LSU). LSU is designed as a holistic measure of network quality, which reflects the structure's ability to form a PBG. LSU captures all the essential features of the PBG size hierarchy: the uniquely-defined states of maximum LSU are the champion diamond and near-champion single network gyroid structures; LSU naturally penalises those networks with complex vertices by calculating tree spatial similarity statistics for inaccessible root-edge alignment permutations and, LSU decreases with increasing structural disorder.

The effect of increasing local self-uniformity can be tracked by plotting a sequence of photonic band structures for our 216-vertex amorphous gyroid networks. In Supplementary Figure 2 we plot band structures for 5 distinct networks with their mean LSU $\overline{\Phi}_{22}$ increasing from left to right. Bands are calculated along the k-space path $\Gamma \rightarrow$X$\rightarrow$M$\rightarrow$R$\rightarrow \Gamma$, where these points refer to the usual high symmetry points of a cubic Brillouin zone. We employ a permittivity contrast of $13:1$ and a dielectric fill fraction of 27.5% (which was found to be approximately optimal for type-2 amorphous gyroids). The bands immediately above and below the largest observable band gap are highlighted in black, and the band gap between them expressed as percentage. We note that the band structures are highly folded because the Brillouin zone of the supercell is artificially small. As a result, band structures are not expected to reflect the band structure of a single network gyroid primitive cell (Supplementary Figure 1) in the limit where $\bar{\Phi}_{22}$ tends to unity (and although the PBG size will tend to the single network gyroid value, it will not be equal to the value in Supplementary Figure 1 due to the difference in filling fraction).

We observe that the density of bands in the region $0.184 < a/\lambda < 0.233$ (the gap for single network gyroid of the same dielectric contrast and fill fraction) is progressively reduced with increasing LSU. This reflects a decreasing density of states ($\rho(\omega)$, DOS) in this region as shown in the evolution of the integrated DOS in Figure 4B. As the LSU increases, the local geometrical environments of the scattering units become more similar. As a result, electromagnetic resonances they support become increasingly degenerate and the frequency region for which transmission is strongly suppressed widens.

Further, we note that a 216-vertex cubic supercell of single network gyroid exhibits its PBG between bands 108 and 109. Indeed a general N-vertex supercell has a PBG from bands $N/2$ to $N/2+1$. This is true also of the largest band gap for our amorphous structures D and E in Supplementary Figure 2. The largest gap in each of band structures A, B and C in Supplementary Figure 2 however falls only in the region of, and not exactly between, bands 108 and 109. For these samples numerous bands exist within the expected band gap region which we associate with defect modes. Such modes are created by geometrical and topological imperfections in the structure of the network. These defect modes crowd the band gap in an unpredictable way, with the result that the gap from bands 108 to 109 is not necessarily the largest gap. To accurately reflect a given network's ability to form a band gap we measured band gap widths as the largest visible gap in the structure.

Supplementary Note 2: PBG Size versus Network Size

We investigated also the evolution of PBG width with increasing network size, as measured by their number of component vertices N. Sets of amorphous gyroid networks were produced for network sizes of 108, 216, 324, 512 and $1,000$ vertices. Next we chose a target spatial similarity of 0.89 and for each network size selected a subset of structures whose $\overline{\Phi}_{22}$s were close to this value. In Supplementary Figure 3B we plot the mean of the means $< \overline{\Phi}_{22} >$ for each subset along with error bars corresponding to the standard deviation of each set of $\overline{\Phi}_{22}$s. We note the y axis scale; the standard deviation of each set is very small and all sets have approximately equal $< \overline{\Phi}_{22} >$.

For each network we calculated the PBG width $\Delta\omega/\omega_0$, and for each set we calculated the mean PBG width $\overline{\Delta\omega/\omega_0}$. In Supplementary Figure 3A we plot the evolution of each set's mean PBG width against network size. The error bars correspond to the standard deviation of each set of PBG widths. We have also marked a least squares linear fit (blue) and a least squares constant fit (green) of the data. We observe that PBG width appears to slowly decrease with increasing network size. This occurs in spite of the approximately equal $< \overline{\Phi}_{22} >$s of each set.

The application of PBG amorphous gyroid structures in optical technologies is contingent on the existence of a gap for arbitrarily large network sizes. In this respect the narrowing of the PBG with increasing network size is less than ideal. However, we observe that our definition of a PBG is very restrictive. Specifically, we define a PBG as a complete absence of bands in the photonic band structure. There exist, however, complimentary definitions of the PBG based on attenuation of transmission and full DOS calculations. We expect that adopting one of these alternative definitions should lead to more consistent PBG characteristics as sample size is increased.

We observe also that a similar analysis has not been performed for photonic amorphous diamond (PAD) networks. We expect that PAD should demonstrate the same decrease in PBG width with increasing network size. This behaviour can be interpreted in two ways:

One is that CRN models of amorphous diamond are not strictly hyperuniform[9] (HPU). We expect the same to be true for equivalently glassy amorphous gyroid networks. A hyperuniform material possesses zero density fluctuations for large sampling volumes. A corollary of this is that the local variation in an HPU material's structure is bounded and that the formation of defect modes, associated with deformation of the local structure, is

suppressed. PAD lacks this characteristic and it is thus not assured that the formation of badly deformed scattering centres will be prevented as network size is increased. As size is increased it is therefore possible that defect modes will be engineered.

The second is that the narrowing of the PBG with increasing network size follows naturally from an interpretation of the LSU distributions as probability distributions of the quality of a scattering centre drawn from a CRN. Consider a typical Φ_{22} distribution for PAD as in Figure 3a. We can associate the most deformed and highly strained scattering centres with the lower tail of the LSU distribution; the probability of a scattering centre with a spatial similarity less than some value α occurring is the fraction of the LSU distribution that is contained in the tail $\Phi_{22} < \alpha$. Consider now doubling the size of a network but preserving the form of its Φ_{22} distribution as best we can. The expected number of scattering centres with qualities less than α should approximately double. These scatterers, on account of their deformations, may support local resonances that are defect-like. We would thus expect that an increase in network size will generate more defect modes that may narrow the PBG.

Supplementary Note 3: Experimental and Simulated Transmission

Experimental data (Fig. 5G) appears noisy in comparison to the FDTD results (Fig. 5H). The experimentally-accessible transmission contrast is limited by noise and horn antenna coupling loss to $\sim$ 35 dB (see the Methods section). As a result, transmission values within and around the PBG approach the noise threshold and appear blurry. Our FDTD simulations achieve a total contrast of $\sim$ 60 dB before bottoming-out; when presented using the same contrast as the experimental data, the PBG thus appears much sharper and uniformly blue (Fig. 5H).

Supplementary Note 4: Brillouin Zone Path for Gyroid Crystals

Band structure is typically calculated along a $\mathbf{k}$-space path which passes through all the high symmetry points of the first Brillouin zone (BZ)[10]. More generally, in order to capture all modes which might couple to an incident plane wave with wavevector $[hkl]$ (expressed in the reciprocal lattice basis), we must compute the band structure along the vector $[hkl]$ until another Γ point is reached. In practice, we need only proceed halfway to the next Γ point due to the time reversal symmetry of the Maxwell equations for lossless media[11,12]. The band structure was hence calculated along a k-space path running from H'→P→ Γ → H → N →P, where all points, except H', refer to the usual high symmetry points of the body-centred cubic (bcc) first BZ. H' lies outside the first BZ along the Γ →P direction, and is exactly halfway to the next Γ point along this axis[12]. An accurate measurement of the stop-gap size along the $[111]$ axis must consider this extended path.

Supplementary Methods

Amorphous Gyroid Simulated Annealing. To generate amorphous gyroid configurations we followed the protocol of Wooten, Winer and Weaire (WWW), incorporating many of the refinements subsequently developed for modelling large amorphous silicon networks[13,14]. The basic process involves the simulated annealing of a random network, which is allowed to explore a multitude of geometric and topological configurations.

The major steps of the WWW algorithm are as follows. First, a random network is altered by the addition of a Stone-Wales defect. This defect alters the topology about two connected vertex points. The network is then relaxed to minimise an energy function; vertex positions are optimised to find the most energy-efficient positions for the given topology. This new state of the network is then evaluated; it is accepted if the energy is found to decrease, otherwise an acceptance probability, P_a is calculated according to

$$P_\text{a} = e^{(E_0 - E_f)/T}, \tag{1}$$

where E_0 and E_f represent the energies of the initial and altered network configurations, respectively, and T is a scaled temperature variable. A random number uniformly distributed on $[0, 1]$ is generated, and the altered configuration is accepted if it is less than P_a. If the altered configuration is accepted, the network is evolved, and the process begins again with the introduction of a new Stone-Wales defect. Otherwise, the network reverts to its original state and a different Stone-Wales defect is tested. The network is driven into a low energy state by gradually reducing the temperature.

In the context of amorphous silicon modelling, the network energy is calculated with a Keating potential. Initially a Keating potential, modified to favour 120° bond angles, was tried. However, networks were not driven into a faithful amorphous gyroid configuration, producing only type-1 networks after $\sim 100,000$ WWW iterations. It is unknown whether dihedral order would have arisen spontaneously, as appears to occur in amorphous diamond CRNs, with further annealing. To accelerate convergence, we introduced a new potential energy function of the form

$$U = \alpha f_1(\{d\}) + \beta f_2(\{\theta\}) + \gamma f_3(\{\phi\}) + \delta f_4(\{\chi\}), \tag{2}$$

where the functions f_1 and f_2 correspond to Keating energy-like terms, penalising deviation from fixed edge length and 120° bond angles, respectively. To this we add the term f_3, which favours dihedral angles ϕ of $arcos(\pm\frac{1}{3})$ as found in single network gyroid, and f_4, which favours configurations in which the three edges about a vertex are all coplanar. Consider two vertices i and j which share a common edge; each has three connections to vertices $[j, i_1, i_2]$ and $[i, j_1, j_2]$, respectively. We express these extra terms f_3 and f_4 as

$$f_3 = \sum_i \sum_j (|\hat{\boldsymbol{n}}_{i_1,i_2} \cdot \hat{\boldsymbol{n}}_{j_1,j_2}| - 1/3)^2 \tag{3}$$

$$f_4 = \sum_i \sum_j (\hat{\boldsymbol{r}}_{ij} \cdot \hat{\boldsymbol{n}}_{i_1,i_2})^2 + (\hat{\boldsymbol{r}}_{ij} \cdot \hat{\boldsymbol{n}}_{j_1,j_2})^2, \tag{4}$$

where $\hat{\boldsymbol{r}}_{ij}$ represents the unit vector from i to j and $\hat{\boldsymbol{n}}_{i_1,i_2}$ the unit normal to the plane containing the vectors $\boldsymbol{r}_{i,i_1}$ and $\boldsymbol{r}_{i,i_2}$. We note that f_3 is minimsed when dihedral planes cut at an angle of $\arccos(\pm 1/3) = 70.53^\circ$ and 109.47°. Ideally we would demand consistent dihedral angles corresponding to either, but not both, of these two values. However, due to difficulties in maintaining consistent vertex labels we were unable to define plane normals $\hat{\boldsymbol{n}}_{i_1,i_2}$ and $\hat{\boldsymbol{n}}_{j_1,j_2}$ such that their scalar products were always positive. We thus take the absolute value $|\hat{\boldsymbol{n}}_{i_1,i_2} \cdot \hat{\boldsymbol{n}}_{j_1,j_2}|$ and accept that mixed dihedral angle centres will be created.

Periodic boundary conditions were employed such that resultant samples could be used in photonic band structure calculations. High-quality amorphous gyroid networks were successfully generated from random seed patterns after around $100,000$ WWW iterations.

Band Gap Calculation and DOS Estimation. Photonic band structures were calculated using MPB[10], the MIT photonic bands software package, in which the eigenmodes of the electromagnetic field are found by plane wave expansion of the Maxwell equations. In all cases a dielectric contrast of $13:1$ was employed. All amorphous gyroid CRNs were constructed from connected dielectric cylinders of equal radius r. A dielectric sphere of radius r was also placed at each vertex point. r was chosen such that the dielectric fill fraction for each CRN was 27.5%. A resolution of 16 mesh points per a unit (the effective single network gyroid primitive cell side length) was used.

For an N vertex supercell of single network gyroid the band gap falls between bands $N/2$ and $N/2+1$. This remains true for the most locally self-uniform of our amorphous gyroid networks, but fails as the disorder is increased. The more disordered networks exhibited multiple small band gaps as a result of defect modes crowding the expected gap region. As a result, the largest band gap did not necessarily fall between bands $N/2$ and $N/2+1$ (Supplementary Fig. 2). In this way we see that measurement of the PBG as a discrete gap between bands can generate noisy data; there is a probability that a large PBG will be interrupted by a defect band, thus substantially reducing the measured gap. Gap widths were thus always measured as the largest observable gap in the band structure so as to reflect as accurately as possible the network's propensity to open a gap.

Fig. 4B introduces a more accurate measure of a structure's PBG forming ability. The PBG for single network gyroid of the same dielectric fill fraction and contrast was determined to fall between $0.1838 < a/\lambda < 0.2327$. We call this the gap region of single network gyroid. We then counted the number of photonic bands in the gap region for each of our 216-vertex networks and plotted this against $\overline{\Phi}_{22}$. The number of bands in the gap region is indicative of how many electromagnetic modes a structure supports in that frequency window. It can be interpreted as an integrated density of states across the gap region of single network gyroid.

FDTD Characterisation of Amorphous Gyroid. The expected transmission versus angle of our ceramic cyindrical amorphous gyroid sample was simulated using a FDTD method. We employ a single-shot method in which sources are placed inside the sample and transmission is recorded in all directions at once[15].

We defined our cylindrical amorphous gyroid sample with a scaling parameter $a = 3.13$ mm, radius $10.3a$ and using the same network design as the printed models. This was positioned at the origin of the FDTD region with its axis oriented along $\hat{\boldsymbol{z}}$. 720 point monitors were positioned every 0.5° in a ring of radius $20a$ in the $z=0$ plane. The electric and magnetic fields were recorded by these monitors and the detected intensity calculated as the magnitude of the Poynting vector at that point. All simulations were performed using a uniform 0.1 mm mesh.

First, the effective permittivity of the amorphous gyroid cylinder was calculated by volume averaging to be $\epsilon_r = 3.44$. The cylindrical sample was deactivated, and the FDTD region filled with a homogenous material of this effective permittivity. A single dipole source was placed at the origin. A background spectrum was recorded for this set-up.

Secondly, the homogenous material and single dipole were discarded and the amorphous gyroid cylinder reactivated. 50 dipole sources, each with a random phase and orientation, were located randomly about the origin within a sphere of radius a. The raw transmission spectrum for this setup was recorded and then normalised through division by 50 times the background intensity.

The method used is a direct probe of the local density of states (LDOS) within the amorphous gyroid network. The power emitted by the dipole sources is strongly suppressed within the band gap by the corresponding zero LDOS. Above the gap, however, emitted power can increase above the corresponding free-space level by an enhancement of the LDOS. Thus, some transmission enhancement (up to a factor of 2) was observed even when the raw transmittance was normalised to the background. A final normalisation was performed by dividing the normalised transmittance by its maximum value.

Supplementary References

1. Maldovan, M. & Thomas, E. Diamond-structured photonic crystals. *Nat. Mater.* **3,** 593–600 (2004).
2. Edagawa, K., Kanoko, S. & Notomi, M. Photonic amorphous diamond structure with a 3D photonic band gap. *Phys. Rev. Lett.* **100,** 013901 (2008).
3. Joannopoulos, J. *et al. Photonic Crystals: Molding The Flow of Light* (Princeton University Press, Princeton, 2011).
4. Men, H., Lee, K. Y. K., Freund, R. M., Peraire, J. & Johnson, S. G. Robust topology optimization of three-dimensional photonic-crystal band-gap structures. *Opt. Express* **22,** 22632–22648 (2014).
5. Maldovan, M., Ullal, C. K., Carter, W. C. & Thomas, E. L. Exploring for 3D photonic bandgap structures in the 11 f.c.c. space groups. *Nat. Mater.* **2,** 664–7 (2003).
6. Edagawa, K. Photonic crystals, amorphous materials, and quasicrystals. *Sci. Technol. Adv. Mater.* **15,** 034805 (2014).
7. Maldovan, M. & Thomas, E. Photonic crystals: six connected dielectric networks with simple cubic symmetry. *J. Opt. Soc. Am. B* **22,** 466–473 (2005).
8. Sunada, T. Crystals that nature might miss creating. *Not. AMS* **55,** 208–215 (2008).
9. Hejna, M., Steinhardt, P. J. & Torquato, S. Nearly hyperuniform network models of amorphous silicon. *Phys. Rev. B* **87,** 245204 (2013).
10. Johnson, S. & Joannopoulos, J. Block-iterative frequency-domain methods for Maxwell's equations in a planewave basis. *Opt. Express* **8,** 173–190 (2001).
11. Saba, M., Wilts, B. D., Hielscher, J. & Schröder-Turk, G. E. Absence of circular polarisation in reflections of butterfly wing scales with chiral Gyroid structure. *Mater. Today Proc.* **1S,** 193–208 (2014).
12. Saba, M. & Schröder-Turk, G. E. Bloch modes and evanescent modes of photonic crystals: weak form solutions based on accurate interface triangulation. *Crystals* **5,** 14–44 (2015).
13. Vink, R. L. C., Barkema, G. T., Stijnman, M. A. & Bisseling, R. H. Towards device-size atomistic models of amorphous silicon. *Phys. Rev. B* **64,** 245214 (2001).
14. Mousseau, N. & Barkema, G. T. Fast bond-transposition algorithms for generating covalent amorphous structures. *Curr. Opin. Solid State Mater. Sci.* **5,** 497–502 (2001).
15. Pollard, M. E. & Parker, G. J. Low-contrast bandgaps of a planar parabolic spiral lattice. *Opt. Lett.* **34,** 2805–7 (2009).
